# Synergistic effects of hip/knee osteoarthritis and comorbidities on mobility and self-care limitations among older adults: Cross-sectional analysis of the Oxford pain, Activity and Lifestyle study

**DOI:** 10.1177/2235042X20974529

**Published:** 2020-12-04

**Authors:** Philippa JA Nicolson, Esther Williamson, Hopin Lee, Alana Morris, Angela Garrett, Maria T Sanchez-Santos, Sarah E Lamb

**Affiliations:** 1 Centre for Rehabilitation Research in Oxford, Nuffield Department of Orthopaedics, Rheumatology and Musculoskeletal Sciences (NDORMS), University of Oxford, UK; 2 School of Medicine and Public Health, University of Newcastle, New South Wales, Australia; 3 College of Medicine and Health, University of Exeter, UK

**Keywords:** Osteoarthritis, comorbidities, synergism, mobility and self-care limitations

## Abstract

**Objective:**

To estimate synergistic effects of hip/knee osteoarthritis (OA) and comorbidities on mobility or self-care limitations among older adults.

**Methods:**

We used baseline, cross-sectional data from the Oxford Pain, Activity and Lifestyle (OPAL) study. Participants were community-dwelling adults aged 65 years or older who completed a postal questionnaire. Participants reported demographic information, hip/knee OA, comorbidities and mobility and self-care limitations. We used modified Poisson regression models to estimate the independent and combined relative risks (RR) of mobility or self-care limitations, the relative excess risk due to interaction (RERI) between hip/knee OA and comorbidities, attributable proportion of the risk due to the interaction and the ratio of the combined effect and the sum of the individual effects, known as the synergy index.

**Results:**

Of the 4,972 participants included, 1,532 (30.8%) had hip/knee OA, and of them 42.9% reported mobility limitations and 8.4% reported self-care limitations. Synergistic effects impacting self-care limitations were observed between hip/knee OA and anxiety (RR: 3.09, 95% Confidence Interval (CI): 2.00 to 4.78; RERI: 0.93, 95% CI: 0.01 to 1.90), and between hip/knee OA and depressive symptoms (RR: 2.71, 95% CI: 1.75 to 4.20; RERI: 0.58, 95% CI: 0.03 to 1.48). The portion of the total RR attributable to this synergism was 30% and 22% respectively.

**Conclusions:**

This study demonstrates that synergism between hip/knee OA and anxiety or depressive symptoms contribute to self-care limitations. These findings highlight the importance of assessing and addressing anxiety or depressive symptoms when managing older adults with hip/knee OA to minimize self-care limitations.

Mobility and self-care limitations have significant personal and societal impact on older people.^1,2^ Limitations in mobility or self-care among older adults are associated with poorer quality of life, future decline in functioning, increased health and social care costs and increased risk of mortality.^3–5^

Hip and/or knee osteoarthritis (OA) is a common condition among older people, and a leading cause of mobility and self-care limitations.^6–8^ Prevalence estimates for self-reported or symptomatic OA range from 6.2% to 12.3% for the hip, and 7.9% to 38% for the knee.^9^ The prevalence of hip/knee OA has a strong association with increasing age and is more common among women than men.^9^ Hip/knee OA has one of the highest rates of comorbidity (68% to 85%) among chronic diseases.^10,11^ The most common conditions among people with hip/knee OA include cardiovascular diseases, depression, type 2 diabetes, hypertension and other sites of musculoskeletal pain.^11–13^

Previous research has shown that the presence of comorbidities in people with hip/knee OA is associated with increased limitations in functional activities such as mobility and self-care.^14^ However, to date the joint effect of OA and comorbidities on mobility and self-care has not been studied. When the joint effect of both factors exceed the sum of their effects alone, this is known as a synergistic effect.^15^ The effect of OA and comorbidities on mobility and self-care limitations may be additive, or some comorbidities may have synergistic effects on mobility and self-care limitations. Identifying these synergistic effects is important to clinical practice, as it enables clinicians to target their often-limited resources to both hip/knee OA and these comorbidities.

For clinicians and researchers managing older adults and designing and testing interventions for people with hip/knee OA, comorbidities present a challenge. Guidelines for the management of hip/knee OA highlight the importance of assessing for, and understanding the effects of, common comorbidities.^16–18^ Exercise is considered the cornerstone of non-surgical management of hip/knee OA and is recommended in all current clinical guidelines.^16–18^ Exercise is effective in reducing mobility and self-care limitations in people with hip/knee OA.^19,20^ Identifying synergistic effects between hip/knee OA and comorbidities would help clinicians such as physical therapists, and researchers, to tailor exercise interventions to target both OA and comorbidities.

This cross-sectional study aimed to test the hypothesis that the joint effect of hip/knee OA and comorbidities on prevalence of mobility and self-care limitations exceeds the sum of their separate effects. We use the phrase ‘hip/knee OA’ throughout this manuscript to refer to people with osteoarthritis of the hip, of the knee, or of both the hip and knee.

## Patients and methods

### Participants

Data for this analysis were identified from baseline responses in the Oxford Pain, Activity and Lifestyle (OPAL) study,^21^ collected between October 2016 and September 2018.

The OPAL study is a prospective longitudinal cohort study of community-dwelling older adults aged 65 years or older recruited from 35 general practices across England. A detailed profile of the cohort is published elsewhere.^21^ Eligible participants were identified from electronic record searches of primary care practice lists which identified a random sample of up to 400 patients per practice (median: 365; range 158–400) for invitation, stratified into two age bands (65 to 74 years and 75 years and over). Individuals were ineligible if they lived in residential care or a nursing home, those with known terminal illness with a life expectancy of less than 6 months, those who presented with severe health or social concerns sufficient to preclude approach, or those considered unable to provide informed consent.

A total of 12,839 patients were contacted by their general practice and invited to take part in the OPAL study. A consent form, patient information leaflet and baseline questionnaire were sent. People who did not return the questionnaire were sent one postal reminder 4 weeks after the original invitation. Among invited participants, 42.1% (N = 5,409) returned the baseline questionnaire and were enrolled in the study.

Participants were eligible for this analysis if they returned baseline questionnaires with no missing data for the pre-specified variables analysed.

### Measurements

All data (with the exception of the Index of Multiple Deprivation which was derived from respondent postcode) were self-reported by participants in the baseline postal questionnaire. Further details of the measures included in the questionnaires is reported in the OPAL cohort profile.^21^

### Demographic information

Demographic information collected included self-reported age, sex, height, weight, smoking status (ever/never) and living arrangement (alone/with others). Body mass index (BMI) was calculated by the researchers, by dividing weight in kilograms by height in metres squared. Socioeconomic factors assessed included self-reported education level (school/higher education), physical demands of their main occupation (very light-light; moderate; strenuous-very strenuous) and the Index of Multiple Deprivation. The Index of Multiple Deprivation (IMD) provides a relative measure of deprivation based on seven domains: income; employment; education, skills and training; health and disability; crime; barriers to housing and services, and living environment.^22^ Individuals are assigned a percentage (0 indicating most deprived to 100 indicating least deprived). For this analysis IMD scores were divided into quintiles from 1 = 20% most deprived to 5 = 20% least deprived across England. Physical activity was measured in response to the question ‘In the past week how many hours a day have you typically spent moving around on your feet?’ from the Rapid Assessment Disuse Index.^23^ Respondents selected from: Less than 1 hour per day; 1 to less than 3 hours per day; 3 to less than 5 hours per day; 5 to less than 7 hours per day and 7 hours or more per day. Low physical activity was defined as less than 3 hours per day.

### Hip and/or knee OA

We used a composite proxy measure of hip/knee OA, derived by including participants who responded ‘Yes’ to both 1) ‘Has your doctor or nurse ever told you that you have arthritis?’, and 2) ‘During the last 6 weeks have you had pain in the following areas of your body? (hip/s; knee/s)’.

### Comorbidities and other sites of musculoskeletal pain

Participants indicated doctor-diagnosed angina or heart troubles; chronic lung disease; diabetes and high blood pressure. We assessed hearing and visual limitations, using two questions from the Tilburg Frailty Indicator (TFI):^24,25^ ‘Do you experience problems in your daily life due to poor hearing’ (Yes/No) and ‘Do you experience problems in your daily life due to poor vision’ (Yes/No). We also assessed anxiety and depression using two further questions from the TFI: ‘Have you felt nervous or anxious during the last month?’ (Sometimes/Yes/No) and ‘Have you felt down during the past month?’ (Sometimes/Yes/No).^24,25^ ‘Sometimes’ and ‘Yes’ responses were combined to create a dichotomous outcome. Both of these questions have been found to correlate positively and significantly with established longer measures of the construct they assess (the Center for Epidemiologic Studies Depression Scale and the Hospital Anxiety and Depression Scale- Anxiety subscale).^24^

Additional musculoskeletal pain sites were identified in response to the question ‘Have you at any time during the last 6 weeks had pain, aching or discomfort in the following areas of your body?’. Sites were selected from: neck; upper back; low back; shoulder; elbow; wrist/hand and ankle/foot.

### Outcomes

We evaluated mobility and self-care limitations using items 1 and 2 of the EQ-5D five-level version (EQ-5D-5L).^26^ The EQ-5D-5L provides a quick and simple patient-reported self-assessment across five dimensions of quality of life (mobility, self-care, usual activities, pain/discomfort and anxiety/depression).^26^ Each dimension is assessed with a single item. Both the mobility and self-care items have demonstrated high correlations with disease specific functional scales such as the WOMAC or Oxford hip and knee scores. (intraclass correlation coefficients (ICCs) for Mobility: 0.674 compared to WOMAC and 0.51 compared to Oxford hip and knee score; Self-care: 0.695 compared to WOMAC and 0.76 compared to Oxford hip and knee score).^27,28^ Both items also demonstrate acceptable reliability in osteoarthritis populations (ICC’s: mobility: 0.61; self-care: 0.76).^28^ The five-level mobility and self-care outcomes were dichotomized as ‘No/slight problems’ or ‘Moderate/severe problems/unable to’.

### Statistical analysis

We analysed data in Stata Statistical Software Release 15.0 (StataCorp LP, College Station, TX) and report the study in accordance with the STROBE (STrengthening the Reporting of OBservational studies in Epidemiology) recommendations.^29^ The analyses were based on participants with complete cases because of the small proportion of questionnaires that had missing data and missingness mechanisms were estimated to be unrelated to the outcome variables after taking into consideration the included covariates.^30^

We used descriptive statistics to present demographics, the presence of comorbidities and mobility and self-care limitations stratified by presence of hip/knee osteoarthritis. We estimated the independent effects of each health condition on prevalence of mobility and self-care limitations using a modified Poisson approach and robust standard errors.^31,32^

We tested the hypothesis that the combined effect of hip/knee OA and comorbidities on prevalence of mobility and self-care exceeds the sum of their separate effects in two steps. We first estimated the combined effects of each comorbidity with hip/knee OA for each outcome. Each model included three dummy (indicator) variables to reflect the three comparisons against the reference category of neither hip/knee OA nor the comorbidity; hip/knee OA and the comorbidity (RR_11_); hip/knee OA without the comorbidity (RR_10_) and the comorbidity without hip/knee OA (RR_01_). We then calculated three measures of synergism according to Rothman’s definitions.^33^ The relative excess risk due to interaction (RERI) is the excess risk for those with both hip/knee OA and the comorbidity that is due to the interaction of the two conditions, above the sum of the effects of each exposure. This is calculated by the following equation: RERI_RR_ = RR_11_ − RR_10_ − RR_01_ + 1. The attributable proportion due to the interaction (AP) is the proportion of the combined RR (RR_11_) that is due to interaction between the two conditions; and the synergy index (SI) is the ratio of the combined effect and the sum of the individual effects.^33^ We used the formulae provided by Andersson et al. to estimate the synergism measures.^34^

The RERI provides the direction of any synergistic effects and the AP and the SI provide information on the magnitude of the effects.^34^ The RERI is considered the primary indicator of the presence of synergistic effects. A RERI of 0 indicates the two conditions are acting independently without interaction. A negative RERI value indicates that the two conditions interact to reduce the risk of the outcome, and a positive value indicates that the conditions interact to increase the risk of the outcome. The AP can range from 0.0 to 1.0, indicating that 0% to 100% of the total RR is due to the synergistic effect. A SI greater than 1.0 indicates that the combined effect of the conditions is greater than the sum of the individual effects. In the extreme case where two conditions have no effect individually but a measurable effect together RERI and SI would approach infinity and the AP would approach 1.0.^33^

We adjusted all models for potential covariates related to both the hip/knee OA and outcome relationship and the comorbidity and outcome relationship: age, sex, BMI, smoking status, living alone, education level, physical demands of occupation, index of multiple deprivation, number of additional musculoskeletal pain sites reported and physical activity level. Collinearity among covariates was assessed using Spearman’s correlation coefficient. A value of 0.40 or higher was considered to indicate high collinearity. None of the variables considered a priori were highly correlated so all were included in the model. Post-hoc analyses were performed stratified by sex, by age category (65–74 years, 75 years and over), to examine if differences in combined effect of comorbidities and hip/knee OA on mobility and self-care limitations, and/or the presence of synergistic effects existed between these sub-groups. To explore the possibility that participants who attend the same GP practice may be more similar to each other than to other participants we conducted an analysis using clustered standard errors where the cluster variable was the GP practice.

## Results

### Characteristics of the participants

**Figure 1.**
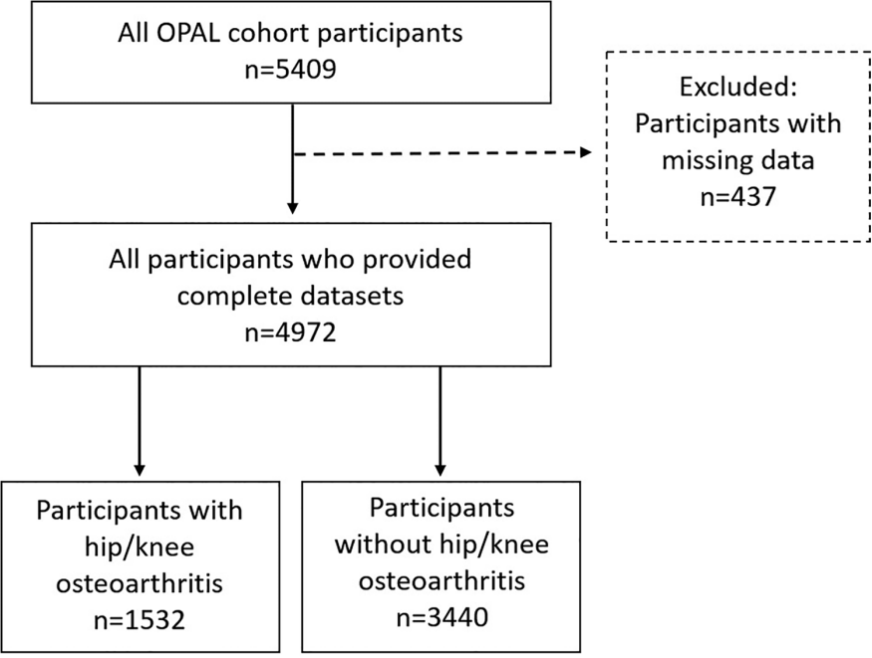
Flow-chart illustrating flow of analysed study participants contributed from the overall Oxford Pain, Activity and Lifestyle (OPAL) cohort.

Of the 5,409 participants enrolled, 437 were excluded because of missing data (Figure 1). Thus, 4,972 participants aged 65 to 100 years were included in the analysis. The majority of excluded participants had missing data for one variable only (339/437; 77.6%), most commonly BMI (224/500; 51.3%). People included in the analysis were younger (mean age: 74.7 (6.7) vs 77.0 (7.2)) and/or less likely to live alone (28.0% vs 39.3%), completed school education only (63.7% vs 73.2%) and less likely to live in the 20% most deprived areas across England (9.8% vs 21.3%) than those excluded from the analysis (Supplementary Table 1).

Table 1 compares demographic characteristics of participants with (n = 1,532/4,972; 30.8%) and without (n = 3,440/4,972; 69.2%) hip/knee OA. Participants with hip/knee OA were more likely to be female, have higher BMI, live alone, work/have worked in a physically demanding occupations, and reside in more deprived areas compared to those without hip/knee OA. They reported lower physical activity levels and pain in three or more sites. The knee alone was most commonly affected (663/1532; 43.3%) among participants with hip/knee OA, followed by both hip and knee joints (550/1532; 35.9%). Participants with hip/knee OA had a higher prevalence of comorbidities overall compared to those without hip/knee OA. They reported significantly higher frequency of high blood pressure, depressive symptoms, anxiety, hearing limitations, angina or heart troubles, vision limitations and diabetes compared to those without hip/knee OA.

**Table 1.** Characteristics of participants with and without hip/knee osteoarthritis from the Oxford Pain, Activity and Lifestyle (OPAL) cohort.

**Characteristic**	**With hip/knee osteoarthritis** **(n = 1532)**	**Without hip/knee osteoarthritis** **(n = 3440)**
Age, mean (SD) years	75.5 (7.0)	74.4 (6.5)
Sex, female (%)	918 (59.9)	1625 (47.2)
Body Mass Index, mean (SD) kg/m^2^	27.7 (5.3)	26.1 (4.6)
Smoking, current/former (%)	792 (51.7)	1708 (49.7)
Living alone (%)	477 (31.1)	917 (26.7)
Education, school only (%)	1036 (67.6)	2131 (62.0)
Physically demanding occupation (%)		
Very light/Light	346 (22.6)	1030 (29.9)
Moderate	734 (47.9)	1597 (46.4)
Strenuous/Very strenuous	452 (29.5)	813 (23.6)
Index of Multiple Deprivation		
1 (20% most deprived in England)	184 (12.0)	305 (8.9)
2 (21–40% most deprived in England)	189 (12.3)	398 (11.6)
3 (41–60% most deprived in England)	323 (21.1)	747 (21.7)
4 (21–40% least deprived in England)	331 (21.6)	757 (22.0)
5 (20% least deprived in England)	505 (32.9)	1233 (35.8)
Physical activity		
Less than 3 hours per day	374 (24.4)	620 (18.0)
No. other musculoskeletal pain sites		
0 painful sites	192 (12.5)	1031 (30.0)
1–2 painful sites	608 (39.7)	1772 (51.5)
3–4 painful sites	480 (31.3)	528 (15.4)
5+ painful sites	252 (16.5)	109 (3.2)
Joint affected		
Hip only, n (%)	319 (20.8)	–
Knee only, n (%)	663 (43.3)	–
Both hip and knee, n (%)	550 (35.9)	–
Other health conditions, n (%)		
High blood pressure	757 (49.4)	1485 (43.2)
Depressive symptoms	655 (42.8)	1069 (31.1)
Anxiety	601 (39.2)	1009 (29.3)
Hearing limitations	455 (29.7)	752 (21.9)
Angina or heart troubles	349 (22.8)	645 (18.8)
Vision limitations	257 (16.8)	366 (10.6)
Diabetes	244 (15.9)	411 (11.9)
Chronic lung disease	147 (9.6)	283 (8.2)
Mobility limitations		
I have no problems in walking about	484 (31.6)	2517 (73.2)
I have slight problems in walking about	488 (31.9)	556 (16.2)
I have moderate problems in walking about	358 (23.4)	274 (8.0)
I have severe problems in walking about	192 (12.5)	84 (2.4)
I am unable to walk about	10 (0.7)	9 (0.3)
Self-care limitations		
I have no problems washing or dressing myself	1228 (80.2)	3225 (93.8)
I have slight problems washing or dressing myself	175 (11.4)	142 (4.1)
I have moderate problems washing or dressing myself	95 (6.2)	52 (1.5)
I have severe problems washing or dressing myself	28 (1.8)	14 (0.4)
I am unable to wash or dress myself	6 (0.4)	7 (0.2)

SD = Standard deviation; IQR = Interquartile range.

### Mobility and self-care limitations

Participants with hip/knee OA more commonly reported mobility and self-care limitations compared to those without. Of note, 36.6% of the cohort with hip/knee OA reported moderate or severe problems with mobility compared to 10.7% of participants without hip/knee OA. The adjusted RR of reporting mobility limitations was 2.08 times greater (95% CI: 1.83 to 2.35) among participants with hip/knee OA compared to those without (Table 2). For self-care limitations 8.4% of the cohort with hip/knee OA reported moderate or severe problems, compared to 2.1% of participants without hip/knee OA. The RR of self-care limitations was 1.57 times greater (95% CI: 1.16 to 2.12) among participants with hip/knee OA.

**Table 2.** Adjusted independent risk ratio of having mobility and self-care limitations in people with hip/knee osteoarthritis and comorbidities.*

	Mobility limitations Functional limitations		Self-care limitations	
Health condition	RR	95% CI	RR	95% CI
Hip and/or knee osteoarthritis	2.08	1.83 to 2.35	1.57	1.16 to 2.12
Hip osteoarthritis only	1.90	1.59 to 2.27	1.08	0.66 to 1.78
Knee osteoarthritis only	2.01	1.73 to 2.32	1.38	0.93 to 2.03
Angina or heart troubles	1.21	1.09 to 1.36	1.02	0.78 to 1.35
Anxiety	1.33	1.19 to 1.48	2.13	1.59 to 2.86
Chronic lung disease	1.41	1.22 to 1.62	1.61	1.16 to 2.24
Depressive symptoms	1.58	1.41 to 1.76	1.80	1.34 to 2.42
Diabetes	1.20	1.06 to 1.36	1.59	1.19 to 2.12
Hearing limitations	1.20	1.07 to 1.34	1.03	0.78 to 1.36
High blood pressure	1.03	0.92 to 1.14	1.01	0.77 to 1.32
Vision limitations	1.24	1.09 to 1.40	1.53	1.12 to 2.08

* Adjusted for age, gender and body mass index, smoking status, living alone, education level, physical demands of occupation, index of multiple deprivation, number of additional musculoskeletal pain sites and physical activity level.

RR = Risk Ratio; CI = Confidence Interval.

### Independent associations between health conditions and mobility and self-care limitations

All health conditions except high blood pressure were independently associated with increased RR of mobility limitations in adjusted regression models (Table 2). Hip/knee OA, anxiety, chronic lung disease, depressive symptoms, diabetes and vision limitations were also independently associated with increased RR of self-care limitations. Among the included conditions hip/knee OA was associated with the highest independent RR of mobility limitation ((RR: 2.08 (95% CI: 1.83 to 2.35)). Anxiety was associated with the highest RR of self-care limitations (RR: 2.13 (95% CI: 1.59 to 2.86)).

### Combined associations and synergistic effects between hip/knee OA and comorbidities on mobility and self-care limitations

The combined effects of hip/knee OA and all included comorbidities were associated with increased risk of mobility and self-care limitations compared to the independent effects of each condition (Table 3). Synergistic effects were observed between hip/knee OA and anxiety, impacting self-care limitations. The RR of reporting self-care limitations was 3.09 times greater (95% CI: 2.00 to 4.78) among participants who reported both hip/knee OA and anxiety compared to those who did not report either condition. The RERI was 0.93 (95% CI: 0.01 to 1.90), indicating that the synergistic effects of hip/knee OA and anxiety on self-care limitations are greater than the sum of the independent effects of each condition. Thirty per cent of the total RR was attributable to this synergism (AP: 0.30 (95% CI: 0.02 to 0.59)). Synergistic effects associated with increased risk of self-care limitations were also observed between hip/knee OA and depressive symptoms. The RR of reporting self-care limitations was 2.7 times greater (95% CI: 1.75 to 4.00) among participants who reported both hip/knee OA and depressive symptoms compared to those who did not report either condition. The RERI between the two conditions was 0.58 (95% CI: 0.03 to 1.48), also indicating that the synergistic effects of hip/knee OA and depressive symptoms on self-care limitations are greater than the sum of the independent effects of each condition. Of the total RR 22% was attributable to this interaction (AP: 0.22 (95% CI: 0.01 to 0.52)). There were no synergistic effects between hip/knee OA and the included comorbidities for the mobility limitations outcome.

**Table 3.** Adjusted relative risks and synergistic effect measures of hip/knee osteoarthritis and comorbidities with regard to the likelihood of mobility and self-care limitations.*

**Variables**	**RR (95% CI)**	**RERI (95% CI)**	**AP (95% CI)**	**SI (95% CI)**
Mobility limitations				
Hip/knee OA + Angina / Heart troubles	2.43 (2.04 to 2.90)	−0.44 (−0.85 to −0.04)	−0.18 (−0.36 to −0.008)	0.76 (0.60 to 0.97)
Hip/knee OA + Anxiety	2.78 (2.33 to 3.32)	−0.48 (−0.92 to −0.05)	−0.17 (−0.33 to −0.01)	0.79 (0.64 to 0.96)
Hip/knee OA + Chronic lung disease	2.52 (2.06 to 3.10)	−0.63 (−1.23 to −0.04)	−0.25 (−0.51 to 0.01)	0.71 (0.51 to 0.98)
Hip/knee OA + Depressive symptoms	3.32 (2.77 to 4.00)	−0.10 (−0.55 to 0.35)	−0.03 (−0.17 to 0.11)	0.96 (0.80 to 1.16)
Hip/knee OA + Diabetes	2.42 (2.04 to 2.88)	−0.02 (−0.43 to 0.39)	−0.01 (−0.18 to 0.16)	0.99 (0.74 to 1.32)
Hip/knee OA + Hearing limitations	2.45 (2.06 to 2.92)	−0.28 (−0.67 to 0.12)	−0.11 (−0.28 to 0.05)	0.84 (0.66 to 1.06)
Hip/knee OA + High blood pressure	2.16 (1.82 to 2.57)	−0.05 (−0.38 to 0.28)	−0.02 (−0.18 to 0.13)	0.96 (0.73 to 1.26)
Hip/knee OA + Vision limitations	2.47 (2.07 to 2.95)	−0.08 (−0.52 to 0.36)	−0.03 (−0.21 to 0.15)	0.95 (0.71 to 1.27)
Self-care limitations				
Hip/knee OA + Angina / Heart troubles	1.59 (1.04 to 2.43)	−0.48 (−1.30 to 0.34)	−0.30 (−0.84 to 0.23)	0.55 (0.22 to 1.40)
Hip/knee OA + Anxiety	3.09 (2.00 to 4.78)	0.93 (0.01 to 1.90)	0.30 (0.02 to 0.59)	1.81 (0.83 to 3.92)
Hip/knee OA + Chronic lung disease	2.27 (1.43 to 3.63)	−0.68 (−2.07 to 0.72)	−0.30 (−0.97 to 0.37)	0.65 (0.28 to 1.52)
Hip/knee OA + Depressive symptoms	2.71 (1.75 to 4.20)	0.58 (0.03 to 1.48)	0.22 (0.01 to 0.52)	1.49 (0.72 to 3.21)
Hip/knee OA + Diabetes	2.43 (1.59 to 3.71)	−0.20 (−1.27 to 0.86)	−0.08 (−0.53 to 0.37)	0.88 (0.44 to 1.74)
Hip/knee OA + Hearing limitations	1.61 (1.06 to 2.43)	−0.22 (−0.99 to 0.54)	−0.14 (−0.62 to 0.34)	0.73 (0.28 to 1.94)
Hip/knee OA + High blood pressure	1.62 (1.06 to 2.48)	−0.15 (−0.86 to 0.56)	−0.09 (−0.52 to 0.34)	0.81 (0.32 to 2.01)
Hip/knee OA + Vision limitations	2.38 (1.52 to 3.74)	−0.30 (−1.41 to 0.82)	−0.12 (−0.61 to 0.36)	0.82 (0.41 to 1.67)

* Adjusted for age, sex, body mass index, smoking status, living alone, education level, physical demands of occupation, Index of Multiple Deprivation, number of additional musculoskeletal pain sites and physical activity level.

OA = Osteoarthritis; RR = Relative Risk; CI = Confidence Interval; RERI = Relative Excess Risk due to Interaction;

AP = Attributable Portion of risk due to interaction; SI = Synergy Index (ratio of the combined effect and the sum of the individual effects).

Post-hoc sensitivity analysis revealed a number of differences in the combined effect of OA and comorbidities on limitations in mobility or self-care between males and females (Supplementary Table 2), and between age categories (Supplementary Table 3), however no synergistic effects were observed. The RR of self-care limitations was higher among females than males when anxiety, chronic lung disease, or depression were reported in addition to hip/knee OA. The RR of mobility limitations was higher among respondents aged 65–74 compared to those aged 75 years or older for depression and hip/knee OA. The RR of self-care limitations was higher among respondents aged 75 years or older compared to those aged 65–74 for angina/heart troubles, anxiety, chronic lung disease, depression, diabetes, hearing limitations and vision limitations. No differences in the results were found in analysis clustered by GP practice.

## Discussion

The objective of this study was to test the hypothesis that the combined effect of hip/knee OA and comorbidities on prevalence of mobility and self-care limitations exceeds the sum of their separate effects. To our knowledge this is the first study to investigate the synergistic effects between hip/knee OA and comorbidities in relation to mobility and self-care limitations among a large cohort of community-dwelling older adults. Among the OPAL cohort who reported hip/knee OA, anxiety and depressive symptoms were two of the most prevalent comorbidities, and both demonstrated synergistic relationships with hip/knee OA, associated with moderate to extreme self-care limitations. The AP for each of these relationships indicates that 22% to 30% of the total observed RR of self-care limitations among people who have hip/knee OA and anxiety, or depressive symptoms, is attributable to interaction between the conditions. We did not observe synergistic effects between any of the included comorbidities and hip/knee OA for mobility limitations.

The prevalence of hip/knee OA observed in our cohort (30.8%) is consistent with large epidemiological studies utilizing self-report measures among older adults.^9,35,36^ Our observation of higher frequency of high blood pressure, depressive symptoms, anxiety, hearing limitations, angina or heart troubles, vision limitations and diabetes among respondents with hip/knee OA compared to those without is also consistent with previous observational studies.^37^ However, the frequency of anxiety (39.2%) or depressive symptoms (42.8%) reported by our cohort with hip/knee OA was higher than the pooled prevalence observed among participants with lower limb OA in a recent systematic review (Depression: 23.0% (95% CI: 16.4–30.2%); Anxiety: 28.2% (95% CI: 23.0–33.8%)).^38^ This may be due to our use of a single self-reported question to assess these conditions. Consistent with previous studies, we observed increased relative risk of mobility limitations in the presence of angina or heart trouble, anxiety, chronic lung disease, depressive symptoms, diabetes, hearing limitations or vision limitations.^7,11,39^ Our finding of higher risks of self-care limitations among females compared to males, and among respondents aged 75 years or older compared to those aged 65–74 for a number of health condition are also consistent with previous observations.^40,41^

Previous studies have not utilized measures of synergistic effects to assess the impact of hip/knee OA and comorbidities on outcomes, precluding direct comparison of our findings in context. However, similar synergistic effects have been observed between depression and other chronic health conditions among older adults. Ho and colleagues observed synergistic effects of comorbidities (defined as any additional chronic medical condition) and depression associated with increased disability and decreased quality of life among older adults in a longitudinal study in Singapore.^42^ They found the associations of comorbidities with increased odds of disability and decreased quality of life were at least threefold stronger among depressed than non-depressed individuals.^42^ Another recent study observed synergistic effects between pain and depression, contributing to increased risk of frailty. The risk of reporting frailty doubled when pain and depression were both present, compared with each condition alone.^43^

We did not observe synergistic effects between hip/knee OA and the included comorbidities associated with mobility limitations. This finding does not suggest that these comorbidities are not associated with increased risk of mobility limitations. Instead it suggests that in this sample the risk of mobility limitations in people with both hip/knee OA and the included comorbidities does not exceed the sum of the risks associated with either condition alone.

We cannot specify the mechanisms which explain the observed synergy between hip/knee OA and anxiety or depressive symptoms associated with self-care limitations. However, OA, anxiety and depressive symptoms share common physiological processes (e.g. inflammation) and risk factors (e.g. physical inactivity).^44^ Chronic low-grade inflammation may trigger a cascade of reactions resulting in the development of a ‘vicious cycle’ of chronic diseases and poor outcomes.^45^ Physical inactivity has been found to exacerbate this ‘vicious cycle’, both through increasing inflammation and poor engagement with self-management strategies.^44^ In addition, older adults with depressive symptoms have been found to have increased perception of functional limitations compared to their objectively measured physical function, which may impact self-reported outcomes.^46^

Our findings have important implications for clinicians, including general practitioners and physical therapists, managing older adults with hip/knee OA. Previous studies have demonstrated that anxiety and depression are often underdiagnosed and undertreated among people with OA.^47,48^ Routine assessment of anxiety and depression should be included for all patients presenting with hip/knee OA. This may be done informally by including questions in assessment, or formally by using a validated questionnaire such as the Hospital Anxiety and Depression Scale (HADS)^49^ or the Depression Anxiety Stress Scale (DASS).^50^ When anxiety or depression is indicated clinicians should refer or signpost for additional mental health assessment and tailored medications. Physical therapy interventions should focus on increasing physical activity and structured, supervised exercise programs as these have been shown to benefit both hip/knee OA and anxiety or depressive symptoms.^19,20,51,52^ Evidence suggests that many types of exercise are beneficial for both hip/knee OA and anxiety or depression, so choice should be guided by patient preference and goals.^19,20,51,53^ Patients with anxiety or depressive symptoms may particularly lack motivation to exercise.^54^ Incorporating principles of behaviour change including social support, self-efficacy, active choices, goal setting and positive reinforcement may be beneficial for maximizing patient participation and adherence to exercise long-term.^55^ Clinicians should also consider referral for psychological therapies.^55,56^

Our study also highlights areas for future research. Studies evaluating these synergistic relationships in a longitudinal design are needed to determine the nature of these effects over time. Randomized controlled trials of interventions targeting both hip/knee OA and anxiety or depression are also needed.

This study has several limitations. The cross-sectional nature of the data does not allow conclusions about causality between hip/knee OA, comorbidities and mobility and self-care limitations. Longitudinal studies are needed to confirm these findings and allow exploration of causality. Differences were observed between included participants and those excluded due to missing data. These differences may impact the generalizability of the results. Data were obtained from a self-reported questionnaire, which may be influenced by misunderstanding or recall. Single questions were used to assess mobility and self-care limitations, and all health conditions which may introduce bias. Duration of symptoms was not accounted for in analyses. Doing so may provide further insight into synergistic effects in sub-groups of patients with chronic vs acute conditions. By combining hip and/or knee OA together we may have introduced bias as hip OA and knee OA may interact differently with comorbidities. Finally, while the cohort has broad participation across England, the results may not be generalizable to other countries.

In conclusion, the results of this study demonstrate that synergism between hip/knee OA and anxiety or depressive symptoms contribute to increased risk of moderate to extreme self-care limitations. These findings highlight the importance of assessing and addressing anxiety and depressive symptoms among older adults with hip/knee OA, to optimize functional outcomes.

## Supplemental material

Nicolson_R2_SuppTables - Synergistic effects of hip/knee osteoarthritis and comorbidities on mobility and self-care limitations among older adults: Cross-sectional analysis of the Oxford pain, Activity and Lifestyle study Click here for additional data file. Nicolson_R2_SuppTables for Synergistic effects of hip/knee osteoarthritis and comorbidities on mobility and self-care limitations among older adults: Cross-sectional analysis of the Oxford pain, Activity and Lifestyle study by Philippa JA Nicolson, Esther Williamson, Hopin Lee, Alana Morris, Angela Garrett, Maria T Sanchez-Santos, Sarah E Lamb and On behalf of the OPAL study team in Journal of Comorbidity
