# Supplementary material for: Synergistic effects of hip/knee osteoarthritis and comorbidities on mobility and self-care limitations among older adults: Cross-sectional analysis of the Oxford pain, Activity and Lifestyle study
Source: J Comorb. 2020 Dec 4;10:2235042X20974529. doi: 10.1177/2235042X20974529 (PMC7720340; doi:10.1177/2235042X20974529)
Supplement: Nicolson_R2_SuppTables - Synergistic effects of hip/knee osteoarthritis and comorbidities on mobility and self-care limitations among older adults: Cross-sectional analysis of the Oxford pain, Activity and Lifestyle study [file Nicolson_R2_SuppTables.docx]

**Supplementary Table 1. Characteristics for complete cases versus excluded cases**

|  | **Complete cases**  **n=4972** | **Incomplete cases (excluded)**  **n=437** | **p-value** |
| --- | --- | --- | --- |
| Age, mean (SD) | 74.7 (6.7) | 77.0 (7.2) | <0.001 |
| Sex, female (%) | 2543 (51.2) | 241 (55.2) | 0.11 |
| BMI, mean (SD) | 26.6 (4.9) | 27.0 (4.5) | 0.10 |
| Smoker / ex-smoker (%) | 2500 (50.3) | 202 (49.6) | 0.80 |
| Living alone (%) | 1394 (28.0) | 161 (39.3) | <0.001 |
| Education level, school only (%) | 3167 (63.7) | 292 (73.2) | <0.001 |
| Physically demanding occupation (%) |  |  | <0.001 |
| Very light / Light | 1376 (27.7) | 70 (17.9) |  |
| Moderate | 2331 (46.9) | 202 (51.5) |  |
| Strenuous / Very strenuous | 1265 (25.4) | 120 (30.6) |  |
| Low physical activity (%) | 994 (20.0) | 105 (26.5) | 0.002 |
| Index of Multiple Deprivation |  |  | <0.001 |
| 1 (20% most deprived) | 489 (9.8) | 93 (21.3) |  |
| 2 | 587 (11.8) | 75 (17.2) |  |
| 3 | 1070 (21.5) | 85 (19.5) |  |
| 4 | 1088 (21.9) | 78 (17.9) |  |
| 5 (20% least deprived) | 1738 (35.0) | 106 (24.3) |  |
|  |  |  |  |
| Hip and/or knee osteoarthritis | 1532 (30.8) | 131 (30.0) | 0.72 |
| Angina or heart troubles | 994 (20.0) | 100 (22.9) | 0.15 |
| Anxiety | 1610 (32.4) | 121 (30.8) | 0.52 |
| Chronic lung disease | 430 (8.7) | 34 (7.8) | 0.53 |
| Depressive symptoms | 1724 (34.7) | 152 (38.4) | 0.14 |
| Diabetes | 655 (13.2) | 82 (18.8) | 0.001 |
| Hearing limitations | 1207 (24.3) | 115 (28.2) | 0.08 |
| High blood pressure | 2242 (45.1) | 217 (49.7) | 0.07 |
| Vision limitations | 623 (12.5) | 81 (19.7) | <0.001 |

**Supplementary Table 2. Adjusted relative risks and relative excess risk due to interaction of the likelihood of mobility and self-care limitations for hip/knee osteoarthritis and comorbidities, stratified by sex.***

|  | Males  n=2429 | | Females  n=2543 | |
| --- | --- | --- | --- | --- |
| **Variables** | **RR (95% CI)** | **RERI (95% CI)** | **RR (95% CI)** | **RERI (95% CI)** |
| Mobility limitations |  |  |  |  |
| Hip/knee OA + Angina / Heart troubles | 2.65 (2.08 – 3.38) | -0.60 (-1.21 – 0.01) | 2.23 (1.73 - 2.88) | -0.50 (-1.09 – 0.09) |
| Hip/knee OA + Anxiety | 2.86 (2.21 – 3.40) | -0.45 (-1.08 – 0.19) | 2.70 (2.06 - 3.53) | -0.48 (-1.10 – 0.13) |
| Hip/knee OA + Chronic lung disease | 2.54 (1.92 – 3.35) | -1.30 (-2.15 – 0.04) | 2.42 (1.80 - 3.25) | -0.09 (-0.89 – 0.71) |
| Hip/knee OA + Depressive symptoms | 3.59 (2.80 – 4.60) | -0.03 (-0.71 – 0.65) | 3.06 (2.34 - 4.01) | -0.04 (-0.63 – 0.55) |
| Hip/knee OA + Diabetes | 2.30 (2.10 – 3.10) | -0.48 (-1.08 – 0.11) | 2.48 (1.94 - 3.17) | 0.16 (-0.44 – 0.77) |
| Hip/knee OA + Hearing limitations | 2.83 (2.21 – 3.62) | -0.41 (-1.03 – 0.21) | 2.12 (1.66 - 2.71) | -0.30 (-0.84 – 0.23) |
| Hip/knee OA + High blood pressure | 2.37 (1.88 – 2.98) | -1.19 (-1.89 - 0.50) | 2.00 (1.54 - 2.59) | -0.17 (-0.63 – 0.30) |
| Hip/knee OA + Vision limitations | 2.81 (2.19 – 3.59) | 0.15 (-0.51 – 0.81) | 2.19 (1.70 - 2.83) | -0.24 (-0.83 – 0.36) |
| Self-care limitations |  |  |  |  |
| Hip/knee OA + Angina / Heart troubles | 1.55 (0.74 – 2.26) | -0.24 (-1.30 – 0.82) | 1.61 (0.89 - 2.92) | -0.61 (-1.91 – 0.69) |
| Hip/knee OA + Anxiety | 2.56 (1.43 – 4.59) | 0.61 (-0.69 – 1.91) | 3.86 (1.96 - 7.58) | 1.27 (-0.226 – 2.80) |
| Hip/knee OA + Chronic lung disease | 1.28 (0.64 – 2.57) | -1.81 (-3.81 – 0.19) | 3.83 (2.12 - 6.90) | 0.62 (-1.65 – 2.88) |
| Hip/knee OA + Depressive symptoms | 2.19 (1.19 – 4.02) | 0.002 (-1.30 – 1.31) | 3.24 (1.70 - 6.20) | 1.15 (-0.14 – 2.43) |
| Hip/knee OA + Diabetes | 2.19 (1.23 – 3.91) | -0.26 (-1.65 – 1.12) | 2.74 (1.50 - 4.99) | 0.26 (-1.45 – 1.98) |
| Hip/knee OA + Hearing limitations | 1.32 (0.72 – 2.43) | -0.30 (-1.36 – 0.76) | 1.90 (1.08 - 3.35) | -0.23 (-1.45 – 1.00) |
| Hip/knee OA + High blood pressure | 1.29 (0.75 – 2.23) | -0.14 (-1.10 – 0.81) | 2.22 (1.11 - 4.42) | -0.51 (-1.87 – 0.86) |
| Hip/knee OA + Vision limitations | 2.79 (1.42 – 5.51) | -0.47 (-2.35 – 1.41) | 2.14 (1.19 – 3.87) | 0.07 (-1.23 – 1.37) |

RR: Relative risk; RERI: Relative excess risk due to interaction

***** Adjusted for age, body mass index, smoking status, living alone, education level, physical demands of occupation, Index of Multiple Deprivation, number of additional musculoskeletal pain sites and physical activity level.

**Supplementary Table 3. Adjusted relative risks and relative excess risk due to interaction of the likelihood of mobility and self-care limitations for hip/knee osteoarthritis and comorbidities, stratified by age category.***

| **Variables** | Aged 65-74  n=2,825 | | Aged 75+  n=2,147 | |
| --- | --- | --- | --- | --- |
|  | **RR (95% CI)** | **RERI (95% CI)** | **RR (95% CI)** | **RERI (95% CI)** |
| Mobility limitations |  |  |  |  |
| Hip/knee OA + Angina / Heart troubles | 2.53 (1.86 - 3.43) | -0.24 (-0.95 – 0.47) | 2.40 (1.95 – 2.96) | -0.43 (-0.91 – 0.05) |
| Hip/knee OA + Anxiety | 3.27 (2.42 - 4.44) | -0.08 (-0.83 – 0.67) | 2.55 (2.06 – 3.16) | -0.55 (-1.06 - 0.03) |
| Hip/knee OA + Chronic lung disease | 2.86 (2.04 - 4.00) | -1.30 (-2.36 - -0.24) | 2.23 (1.72 – 2.89) | -0.25 (-0.94 – 0.43) |
| Hip/knee OA + Depressive symptoms | 4.07 (2.98 - 5.57) | 0.28 (-0.58 – 1.13) | 2.90 (2.34 – 3.59) | -0.20 (-0.70 – 0.31) |
| Hip/knee OA + Diabetes | 2.81 (2.09 - 3.78) | -0.001 (-0.71 – 0.71) | 2.16 (1.75 - 2.66) | -0.06 (-0.55 – 0.42) |
| Hip/knee OA + Hearing limitations | 2.56 (1.92 - 3.41) | 0.17 (-0.51 – 0.85) | 2.41 (1.94 - 2.99) | -0.40 (-0.88 – 0.08) |
| Hip/knee OA + High blood pressure | 2.53 (1.88 - 3.39) | -0.35 (-1.04 – 0.33) | 1.88 (1.53 - 2.32) | 0.04 (-0.31 – 0.40) |
| Hip/knee OA + Vision limitations | 2.54 (1.87 - 3.47) | 0.12 (-0.66 – 0.90) | 2.45 (1.99 - 3.02) | -0.09 (-0.60 – 0.43) |
| Self-care limitations |  |  |  |  |
| Hip/knee OA + Angina / Heart troubles | 1.11 (0.64 - 1.94) | -0.15 (-0.96 – 0.67) | 2.50 (1.30 – 4.80) | -0.96 (-2.66 – 0.73) |
| Hip/knee OA + Anxiety | 2.08 (1.18 - 3.67) | 0.73 (-0.23 – 1.67) | 4.65 (2.43 – 8.88) | 1.73 (-0.21 – 3.66) |
| Hip/knee OA + Chronic lung disease | 1.59 (0.86 - 2.94) | -1.61 (-3.43 – 0.20) | 3.24 (1.68 – 6.25) | 1.04 (-1.15 – 3.22) |
| Hip/knee OA + Depressive symptoms | 2.10 (1.17 - 3.78) | 0.58 (-0.43 – 1.59) | 3.51 (1.89 – 6.53) | 1.04 (-0.42 – 2.49) |
| Hip/knee OA + Diabetes | 1.90 (1.07 - 3.38) | -0.31 (-1.44 – 0.84) | 3.18 (1.71 - 5.92) | 0.19 (-1.73 – 2.12) |
| Hip/knee OA + Hearing limitations | 1.08 (0.64 - 1.81) | -0.11 (-0.97 – 0.75) | 2.51 (1.28 - 4.95) | -0.71 (-2.33 – 0.92) |
| Hip/knee OA + High blood pressure | 1.41 (0.79 - 2.54) | -1.34 (-2.79 – 0.11) | 1.79 (1.00 - 3.23) | 0.69 (-0.12 – 1.49) |
| Hip/knee OA + Vision limitations | 1.61 (0.90 - 2.89) | -0.53 (-1.89 – 0.82) | 3.33 (1.79 - 6.20) | 0.25 (-1.60 – 2.10) |

RR: Relative risk; RERI: Relative excess risk due to interaction

***** Adjusted for age, body mass index, smoking status, living alone, education level, physical demands of occupation, Index of Multiple Deprivation, number of additional musculoskeletal pain sites and physical activity level.
